# Global longitudinal strain as a predictor of outcomes in chronic Chagas´ cardiomyopathy

**DOI:** 10.1371/journal.pntd.0012941

**Published:** 2025-08-06

**Authors:** Minna Moreira Dias Romano, Henrique Turin Moreira, Fawaz Alenezi, Joseph Kisslo, Bernardo Lombo Lievano, André Schmidt, Benedito Carlos Maciel, José Antônio Marin-Neto, Eric J. Velazquez

**Affiliations:** 1 Cardiology Center of the Medical School of Ribeirao Preto, University of São Paulo (USP), São Paulo, Brazil; 2 Duke Medicine School, Durham, North Carolina, United States of America; 3 Cardiovascular Medicine, Yale School of Medicine, New Haven, Connecticut, United States of America; University of North Carolina at Chapel Hill School of Medicine, UNITED STATES OF AMERICA

## Abstract

**Introduction:**

Chronic Chagas cardiomyopathy (CCC) is associated with a high incidence of cardiovascular events. Global longitudinal strain (GLS) is useful in predicting adverse cardiovascular outcomes in several cardiomyopathies. However, its prognostic value in CCC is not well established.

**Methods:**

This study was a retrospective echocardiography analysis with prospective follow-up of CCC subjects. GLS was defined as the average of three apical peak longitudinal strain measurements of the LV using vendor-independent software. GLS groups were defined according to tertiles: (1) GLS ≤ -18.4%, (2) GLS > -18.4% and <-13.8%, and (3) GLS ≥ -13.8%. The primary outcome was a composite of death, hospitalization, sustained ventricular tachycardia (SVT), new heart failure, any systemic embolism, hospitalization, reverted cardiac arrest and cardiac heart transplantation.

**Results:**

GLS was obtained in 77 subjects, 50.6% were males and the mean age was 56 ± 15 years. There were 6.49% losses of follow-up and the mean LVEF was 51 ± 14%. After a follow-up period of 35 ± 19 months (2.9 y), 33 subjects reached the composite outcome. Death and hospitalization were the most frequent outcomes (n = 9), followed by new heart failure (n = 6), embolism (n = 6), and SVT (n = 3). The GLS ≥ -13.8% was associated with a worse prognosis when compared with the other tertile GLS groups (log rank-p-value = 0.001 for both comparisons). On the multivariate Cox proportional hazard model, adjusting for age, gender, and LVEF, GLS was an independent predictor of outcomes with an HR of 1.20 (CI = 1.05-1.38;p = 0.008).

**Conclusion:**

Left ventricle GLS is an independent predictor of cardiovascular outcomes in patients with CCC. GLS may be an important tool for Chagas disease risk stratification, independent of LVEF.

## Author summary

Chronic Chagas Cardiomyopathy (CCC) is still a tough consequence of a parasitic infection from *T. cruzi* and may present with a high incidence of cardiovascular events in a portion of infected patients. Mortality may be secondary to a pleomorphic cardiovascular presentation, varying from ventricular arrhythmias, heart failure, or even thromboembolic events, such as stroke. However, identifying who will be these patients at high risk is still a challenge. There is a non-invasive tool from a very available cardiac examination, the echocardiography, named *Speckle Tracking*, measuring myocardial deformation (strain) with one variable named global longitudinal strain (GLS). This variable is a predictor of myocardial fibrosis and related to prognosis in several cardiac conditions. However, the prognostic value of Global Longitudinal Strain (GLS) in CCC is not well established.

In this prospective analysis of CCC, patients were separated into tertiles of GLS in groups: (1) GLS ≤ -18.4%, (2) GLS > -18.4% and <-13.8%, and (3) GLS ≥ -13.8%. We studied the primary outcome as a composite of death, hospitalization, sustained ventricular tachycardia (SVT), new heart failure, and embolic events. GLS was an independent predictor of outcomes adjusted for age, gender, and left ventricular ejection fraction in patients with CCC.

## Introduction

Chronic Chagas cardiomyopathy (CCC) is the most common manifestation of Chagas disease (CD), a parasitic infectious disease secondary to Trypanosoma cruzi (*T cruzi)* infection. CCC is also the most ominous form of CD, with mortality rates greater than 10% a year in high-risk cases, with sudden death being a definite hallmark of the disease [1]. Deaths can be secondary to heart failure manifestation but also occur as sudden deaths. The morbidity of CCC is also high, including outcomes such as arrhythmias, heart failure, stroke, and other embolic events [2,3]. These pleomorphic clinical presentations, added to the lifetime-long evolution of the disease (2–3 decades of evolution to CCC), constitute a challenge to risk prediction in these patients [4].

Not only in ischemic but also in several etiologies of non-ischemic cardiomyopathies, including CD, impaired left ventricle systolic function, evaluated as ejection fraction (LVEF), is consistently found to be an independent predictor of mortality [5–8]. However, LVEF may be influenced by several intrinsic factors, including loading conditions and geometric assumptions (extrapolation to measured areas to volumes based on the geometry of LV). Bi-dimensional speckle tracking echocardiography (STE) has evolved as a recent method that measures myocardial deformation in any geometric direction with less influence on load conditions. Thus, STE-derived global longitudinal strain (GLS) has been proven to be more sensitive than LVEF in detecting early myocardial damage and predicting clinical outcomes in non-ischemic cardiomyopathies [9–11].

Previous studies of patients with CD tried to identify clinical and complementary diagnostic tools as predictors of mortality and other outcomes [12–14]. One of the proposed and tested scores, the Rassi Score, combining six parameters including sex, functional class of heart failure symptoms, non-sustained ventricular tachycardia (NSVT), cardiomegaly on chest X-ray, LVEF, and ECG low voltage is a good predictor of outcomes in CCC [1]. Several of these parameters are indirect markers of ventricular dysfunction and the use of STE as a more sensitive parameter and direct marker of early myocardial damage could simplify risk prediction in CCC. The strength of STE in predicting clinical outcomes in CCC is not well established [15]. The present study prospectively evaluated biventricular STE parameters in a cohort of CCC patients and tested LV GLS as a predictor of outcomes.

## Methods

### Ethics statement

The institutional research ethics committee approved the study protocol (process no 4913/2010), which was conducted in accordance with the Helsinki Declaration. Written informed consent was obtained from all participants.

### Study design and population

The study was designed as a retrospective inclusion and an observational prospective cohort. A population of consecutive CCC subjects from a tertiary referral center (Chagas clinics oat the Clinical Hospital of Medical School of Ribeirão Preto- São Paulo University) who have had an echocardiography examination archived in DICOM form, from 2011 to 2014 was selected. Additional inclusion criteria were age > 18y and serological positivity for CD confirmed by at least two different methods (ELISA, indirect immunofluorescence, indirect hemagglutination). Patients were excluded if they had other cardiomyopathies, obstructive coronary disease, severe cardiac valve disease, or any systemic disease with potential effects on LV function. All participants underwent standard 12-lead electrocardiography (ECG), Holter, chest X-ray, and echocardiography. Patients with no ECG or echocardiography abnormalities, who would be classified as an Indeterminate form of Chagas disease, were excluded from the analysis.

### Echocardiography

All individuals underwent transthoracic echocardiography with ultrasound equipment capable of exporting images in Digital Imaging and Communications in Medicine (DICOM) form (Vivid E9 GE Healthcare, Horten, Norway; HD11 or Envisor, Philips Medical Systems, Andover, MA) using a phased-array transducer of 1.5–4.6 MHz. The exams were recorded in raw data in an institutional PACS. Acquisition and reading protocols followed guidelines previously published. Briefly, individuals were examined in left lateral and dorsal supine decubitus using conventional parasternal, apical, and subcostal views. Images were acquired with simultaneous ECG signal recording during quiet respiration. At least three entire cardiac cycles were retrospectively recorded, increasing to five entire cardiac cycles in the presence of cardiac arrhythmia. Images were transferred to a central Lab., after being anonymized, to a workstation to access software independent analyzer (Image Arena 2D Cardiac performance Analysis, V 1.2 TomTec Imaging Systems, Germany).

Conventional measurements of left ventricle end-diastolic (LVEDV) and end-systolic (LVESV) and LV ejection fraction (LVEF) were performed from bidimensional LV images using Simpson’s rule as an average of 3 cycles measurements. The right ventricle fractional area was measured in RV dedicated projection at a 4-chamber view using Simpson’s rule. The LV myocardium was analyzed in four-chamber, two-chamber, and three-chamber views. In each projection, the endocardial border was manually traced in end systole and the software automatically traced a region of interest including the entire myocardium. The integrity of tracking was automatically determined and visually verified by an observer in each LV segment. End systolic time was defined automatically by the software with the minimal volume point at the volumetric curve of the LV. Myocardial peak systolic strain (LV GLS) was measured by software and averaged through the 17-segment model to generate a global longitudinal strain (GLS) value. Similarly, right ventricle STE parameters such as free wall (RV GLS__free wall_) and RV GLS__global_ from apical 4-chamber (RV GLS 4c) view were tracked by strain software (TomTec) with RV dedicated tool. Patients with more than 2 LV or 1 RV segments recognized as bad tracking quality were excluded. All measurements were performed by a single experienced observer. As a default of this software (EchoPac), measurements of STE deformation parameters represent mesomyocardial and not endocardial deformation per se. A randomly selected subset of patients was reevaluated by a second experienced evaluator, blinded to the first measurements, to test interobserver variability.

### Clinical data

For all study subjects enrolled, we collected the following data: age, gender, NYHA (New York Heart Association) functional class, prevalence of hypertension, *diabetes mellitus*, smoking, dyslipidemia, systolic blood pressure (SBP), diastolic blood pressure (DBP) and heart rate (HR) at the time of echocardiography. In Chagas out-patient clinics, patients used to be evaluated as clinical indication, varying from 3-4 months if clinically stable or closer when preset a change in symptoms and echocardiography examinations are also indicated clinically when needed, which used to happen in 1–2 years intervals. Electrocardiographic alterations such as right bundle branch block (RBBB), left bundle branch block (LBBB), left anterior-fascicular block (LAFB), and low QRS voltage and presence of cardiomegaly by the X-ray chest examination. SVT and NSVT from Holter reports in a time range of 6 months to echocardiography were collected. The Rassi score clinical prognostic scale was also recorded in subcategories of low, intermedium or high risk.

### Outcomes

A prospective follow-up included patient clinical consultations and also information on medical records, phone calls, and access to death reports when non-documented in-hospital until the date of December 2017. The primary endpoint was the first occurrence of a composite outcome, which included death, hospitalization, sustained ventricular tachycardia (SVT), development of new-onset heart failure, stroke or any systemic embolism, resuscitated cardiac arrest or cardiac transplantation. The date of the last follow-up was defined as the date of the first outcome or the date of the last clinical visit at a tertiary clinical general hospital, when data were censored during the analysis.

### Statistical analysis

Continuous distribution data were expressed as mean ± standard deviation; categorical data were expressed as absolute and percentage values. Unadjusted Kaplan –Meier curves and log-rank test were used to evaluate survival distributions according to GLS tertiles. Multivariate Cox proportional-hazard models were performed to assess the relationship of GLS (and of 5% of absolute change in GLS) with the composite endpoint, including age, gender, and LVEF as covariates. The significance level was defined as P < 0.05. All statistical analyses were performed using Stata 14.0 (StataCorp, College Station, TX).

### Reproducibility

STE parameters were tested for reproducibility in a subset of 15 subjects randomly selected. For intra-observer variability, a re-reading was performed at least one month after the first reading, blinded to the previous one. For inter-observer variability, a second experienced echocardiographist physician performed analyses using the same technique previously described, also blinded to both the first reader analysis. Results were compared with an intra-class correlation coefficient (ICC) to assess absolute agreement and a coefficient of variation (CV) determined by the standard deviation divided by the mean and expressed as a percentage.

The corresponding author has full access to all the data from the study and takes responsibility for its integrity and the data analysis

## Results

The study population consisted of 77 CCC outpatients from a tertiary referral public-level hospital scanned with echocardiography from 2011-2014 and who had archived images with STE acquisition protocol. The participants were all outpatients. Follow-up time was of 35 ± 19 months (2.9 y). There was a 6.49% loss of follow-up. The population consisted of 50.6% male, the median age was of 56 ± 15 years at the beginning of the study, and 68.83% in NYHA I or II. Patients presented in stages B1 (11%), B2 (67%) and C (22%) and no one in stage D. Patients were well prescribed to angiotensin inhibitors (ACEi in 98%) and beta blockers (B-Block in 78%). None of these patients had received antiparasitic treatment before. ECG’s most prevalent alterations were RBBB in 41.55%, AF in 11.68%, and RBBB+LAFB in 29.87%. The mean LVEF was 51.73 ± 13.93 and GLS -15.5 ± 4.85 (Table 1). None of the patients had received a cardio defibrillator implant during the study.

**Table 1:** Cohort population characteristics.

Demographic and clinical	n = 77
Age	55.18 ± 14.3
Male sex	39 (50.6%)
NYHA class I or II	53 (68.83%)
NYHA class III	17 (22.07%)
Hypertension	6 (7.79%)
Diabetes Mellitus	4 (5.19%)
Smoking	27 (35.06%)
Dyslipidemia	26 (33.76%)
SBP	113.0 ± 22
DBP	66 ± 11.1
HR	65.7 ± 12.4
**Medication use**	
B-Block	60 (78%)
ACEi	75 (98%)
Amiodarone	17 (22%)
**ECG alterations**	
Atrial fibrilation	9 (11.68%)
RBBB	32 (41.55%)
LBBB	8 (10.38%)
LAFB	33 (41.0%)
RBBB+ LAFB	31 (42.85%)
Low QRS voltage	11 (14.28%)
**Chest Xray**	
CTI > 50% on chest X-rays	29 (37.66%)
**Echocardiography**	
LV EDV	124.7 ± 58.6
LV ESV	72.4 ± 52
LVEF	51.7 ± 13.9
RV FAC	41.6 ± 10.8
**LV GLS**	-15.5 ± 4.9
**Rassi score**	7 ± 4
Low	31 (40.26%)
Intermediate	32 (41.56%)
High	14 (18.18%)

NYHA = New York heart association; SBP = systolic blood pressure; DBP = diastolic blood pressure; HR = heart rate; RBBB = right bundle branch block; LBBB = left bundle branch block; LAFB = left anterior bundle branch block; LV = left ventricle; CTI = cardiothoracic index; EDV = end-diastolic volume; ESD = end-systolic volume; LVEF = left ventricle ejection fraction; RV = right ventricle; FAC = fractional area change; GLS = global longitudinal strain.

Death (n = 9, cardiovascular cause) and CV-related hospitalization (n = 9) were the most prevalent isolated outcomes, followed by the development of new-onset HF (n = 6), systemic embolism (including stroke) (n = 6), and SVT (n = 6). No subjects presented resuscitated cardiac arrest or heart transplantation during the time of follow-up. More than one component of composite outcomes occurred in 33 participants during observation follow-up.

Univariate analysis identified measurements of myocardial deformation of LV GLS (HR 1.13, CI 1.07-1.21, p < 0.0001) and LVEF (HR 0.96, CI 0.94-0.98, p 0.003) as significantly related to outcomes. RV GLS (Free wall) (HR 1.01, CI 0.96-1.06) was not significantly related to outcomes (Table 2).

**Table 2:** Relationship between Strain Echocardiography variables and outcomes in patients with Chagas disease.

	Follow up			Composite Outcome (n = 33)			
		35 ± 19 months					
		Univariate		p	Multivariate (HR)		p
		HR	95% CI		HR	95% CI	
**LV GLS**		1.13*	(1.07-1.21)	<0.001	1.20*	(1.05-1.38)	0.008
**RV GLS**_**(Free Wall)**_		1.01	(0.96-1.06)	0.686			
**Age**		1.00	(0.82-3.31)	0.285			
**Gender (male vs female)**		1.65	(0.82-3.31)	0.153			
**LVEF**		0.96*	(0.94-0.98)	0.033	1.02	(0.97-1.07)	0.339

Multivariate analysis adjusted GLS for age, gender, and LVEF, and LVEF for GLS, age and gender. Composite outcome: Death+hospitalization +heart failure+SVT+embolism or stroke.

LV GLS_ left ventricle mesocardial strain; RV GLS (Free wall)= right ventricle global longitudinal strain from free wall. LVEF = left ventricle ejection fraction. HR = Hazard ratio; CI = confidence interval. * = p < 0.05

Multivariate analysis was conducted with a model where deformation strain variables (GLS) were adjusted for age, gender, and LVEF. LV GLS was a predictor of composite outcomes independent of age, gender, and LVEF. When death was analyzed as an individual outcome component, LV GLS was also significantly related to the outcome (HR = 1.18, CI 1.05-1.33, p 0.005) even when adjusted for age and gender, S1 Table.

**Fig 1:**
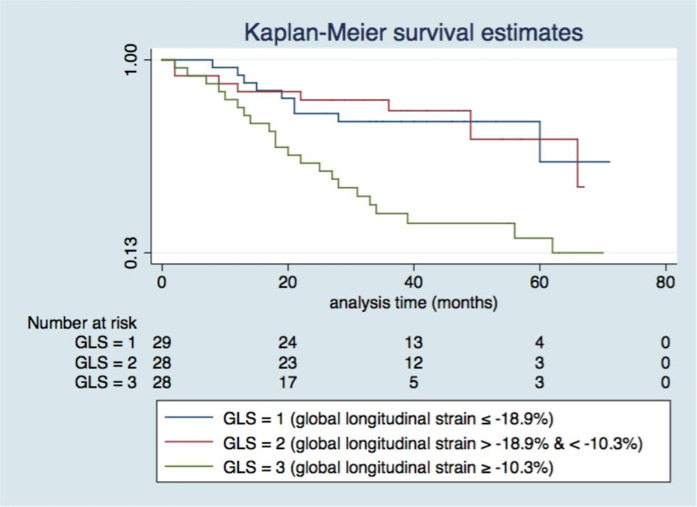
Event-free survival curves of Chagas Chronic Cardiomyopathy (CCC) cohort based on GLS tertiles. Log-rank p value (GLS 1 x GLS 2 and GLS 3) < 0.001.

When the population was separated by tertiles of LV GLS, event-free rates were significantly different. LV GLS 1 ≤ -18.4%, LV GLS 2 > -18.4 < -13.8% and LV GLS 3 ≥ -13.8%, LogRank < 0.001 (Fig 1).

## Reproducibility

Values for intra-observer variability of GLS and LVEF were assessed by intra-class correlation coefficient (ICC) as follows: 0.99 and 0.98. ICC for inter-observer variability of GLS and LVEF were 0.99 and 0.96.

## Discussion

Our study demonstrates that LV myocardial systolic deformation, measured as GLS by advanced echocardiography, is an independent predictor of a composite of clinically relevant hard outcomes in CCC outclinic patients. As far as we know, although GLS has been demonstrated as an independent predictor of outcomes in several non-ischemic cardiomyopathies, some controversies about its predictive power in CCC remain. The present findings also demonstrated that GLS is an independent predictor of death when adjusted for age, gender and LVEF. Moreover, GLS ≥ -13.8% was significantly related to outcomes compared to values ≤ -18.4% and between – 18.4% and -13.8%.

CCC is defined as a cardiac compromise related to chronic *T. cruzi* infection, demonstrated by any electrical and/or structural heart alteration. Alterations are classically demonstrated by ECG, Chest X-ray, and or echocardiography [16]. Mortality in CCC phase is variable between individuals, reaching up to 10% per year in some cohorts [17], but is not the solely adverse outcome. Patients also suffer significantly from clinical heart failure, arrhythmias, sudden death, and thrombo-embolic events such as stroke and pulmonary embolia [18]. Apical aneurysms are common and may be one of the contributors to outcomes related to a cardiac source of embolus. CCC per se is one of the most frequently related causes of ischemic cerebral events in endemic regions such as Latin America [19]. Our cohort of CCC patients presented conspicuous signs of cardiac damage, including ECG alterations in more than 42% (low voltage in 14.28%), cardiomegaly by the X-Ray in 37%, although LVEF was preserved in most of them (51.73 ± 13.93%). A significant proportion of patients were classified as stage B1 or B2 of HF [18], and only 22.07% presented NYHA functional class III; all of them were outpatients. Even considering these aspects, through the 35 ± 19 months (2.9 years) of follow-up, the cumulative mortality rate was 11.68%. The combined outcome happened in 42.85% of the studied population during the time of follow-up. Predicting death and outcomes in such a pleomorphic presentation disease is still a challenge.

Previous studies were able to identify and validate not only isolated but, most importantly, combined predictors of morbi-mortality in specific populations of Chagas´ disease patients [20,21]. Rassi score of risk for CCC [1] tested and validated a score including NYHA functional class, Chest X-ray cardiomegaly, LV alterations by conventional echocardiography [22], NSVT, low voltage ECG, and male gender as good predictors of death. Our cohort presented a medium Rassi score of 7, merging the low to intermediate risk. It is a representative example of a population with a low proportion of symptoms of HF, with a mainly preserved LVEF, but even considering this, with a mortality rate superior to 10% through a follow-up of less than 3 years. Considering the known late-stage characteristic of reduction of LVEF as a marker of myocardial dysfunction, it is comprehensible to seek more sensitive [23] and still low-cost tools to detect myocardial damage and preview clinical outcomes in CCC, such as STE [15,24].

It was already demonstrated that GLS is reduced in CCC with various LV segmental compromises and that they were related not only to global but also to LV regional fibrosis detected by cardiac magnetic resonance (CMR) [25,26]. Indeed, regional longitudinal deformation compromise is present in the very early stages of Chagas disease, such as in some patients at the Indeterminate form, even when no fibrosis is detected by CMR [25]. Also, the prognostic value of fibrosis quantification by CMR in patients with CD was already demonstrated [27]. Some studies also showed that GLS may predict evolution from the Indeterminate form of Chagas disease to CCC [28]. Few previous studies had also demonstrated a prognostic power of STE measurements using different outcomes and in different geographical regions [29]. We also know there are differences in pathogenicity of strains related to different geographic regions of Latin-America [30]. Also, different from our population, other studies included a significant proportion of advanced stages of disease, as NYHA FC IV [29], or even a more heterogeneous population of CD, including Indeterminate Form [31] patients which may interfere with the incidence of adverse outcomes. Other studies demonstrated other still not so applicable measurements of STE, such as GCS [31], as better predictors of outcomes than GLS. So, there is still an interest to test STE as a predictor of outcomes and mortality in CCC [32–34]. A recent study from Saraiva et al. [35] included a wide population of CD patients, not only with CCC but also in other forms of the disease, and they found no evidence of GLS as a predictor of death, even through a long follow-up time of 6.5 ± 2.7 years. Measuring endocardial but not mesocardial GLS may be one of the explanations for this lack of relationship. Chagas cardiomyopathy has a pattern of heterogeneous transmural or mesomyocardial fibrosis substitution when tissue is characterized by CMR [27]. In our study, we measured mesocardial GLS, with the caution to strictly determine it as a systolic peak and not a global peak, even if using a non-vendor dedicated strain software. Using non-vendor-dedicated strain measurement software allows for the determination of measurements independent of machine vendors. It enhances the possibility of using it in any archived type of echocardiographic images with a very good feasibility. Another previous study compared CCC with a group of patients with dilated non-ischemic cardiomyopathy. It demonstrated that, after adjustment for LVEF, there were no differences in STE values between CCC and patients with dilated cardiomyopathies. In that study, after a follow-up time of 18.2 months, the risk of cardiac events evaluated as a composite outcome increased significantly in patients with GLS > - 12% (log-rank p = 0.035).

Echocardiography deformation tools can also measure left atria function, and it was already demonstrated that CCC patients have compromise in both conduit and reservoir LA functions and that peak negative global LA strain function is also an independent predictor of outcomes [36–40]. Our study didn’t test the predictive value of atria strain parameters. CCC patients also may present an isolated or secondary commitment of RV geometry and function, and, although evaluating RV function has limitations when using conventional echocardiographic tools [41], it can be evaluated by STE, with a very good correlation to CMR parameters in CD patients [42]. Our study also evaluated RV GLS but did not find a positive relationship between RV GLS free-wall to outcomes in this cohort of CCC patients.

### Study limitations

Our study has several limitations. Although we have selected only chronic outpatients and not those in other forms of the disease, as in previous studies, which has the advantage of being a more strict population with similar clinical characteristics, the number of patients was limited to those with analyzable archived images to perform STE measurements, as it was a retrospective image collection. It is an aspect related to our limited sample size, but still enough to present significant composite outcomes during the study time. Also, data about the NST of this population was not considered in risk prediction tests. Another important consideration is that this study was not designed to compare STE values with other classical predictors of outcomes in CD, other than age, gender and LVEF or other Rassi score components, and to perform this analysis, sample size should be enhanced.

Applicability: as echocardiography is a very well available and relatively low cost method, even with new technologies as Speckle Tracking software, the use of GLS may be applied as a prognostic tool even in low income countries, where the prevalence of CCC is high. Studies planned to add GLS to other consolidated risk scores in CCC will need to be performed first and to be tested in endemic populations.

## Conclusions

Left ventricle GLS, as evaluated by STE, is an independent significant predictor of hard clinical outcomes in CCC outclinic patients. GLS was also an independent predictor of death when adjusted for age and gender in this cohort of patients.

## Supporting information

S1 Table Relationship between Strain Echo variables and Death in patients with Chagas disease. Legends: LV = left ventricle; RV = right ventricle; GLS = global longitudinal Strain; LVEF = left ventricle ejection fraction. Multivariate analysis in a model adjusted for age and gender. * = p < 0.05.
(XLSX)
